# Supplementary material for: The effect of explicit convection on simulated malaria transmission across Africa
Source: PLoS One. 2024 Apr 16;19(4):e0297744. doi: 10.1371/journal.pone.0297744 (PMC11020401; doi:10.1371/journal.pone.0297744)
Supplement: S1 File — The supporting information consists of: detailed descriptions of utilised malaria models (section S1); analysis supporting the choice of observational products (section S2); and simulated atmospheric biases of historical explicit- and parameterised-convection climate integrations (section S3). The document also contains supporting figures; captions of these are below. (PDF) [file pone.0297744.s001.pdf]

Supporting information

S1 Malaria model descriptions

S1.1 LMM

For a compartmental infectious disease model, an expression for the basic reproduction number, R_0 as a function of model parameters is determined by considering the steady-state conditions of the governing differential equations. R_0 therefore gives an indication of the suitability of conditions, as described by the model parameters, for sustained or increasing disease transmission. For LMM, R_0 is given by equation 1 [1]:

$$R_0 = \frac{ma^2bc}{\mu r} exp(-\mu\tau_y - r\tau_x) \tag{1}$$

where the biting rate (a; day^{-1}); mosquito mortality (μ; 0 to 1); sporogonic cycle length (τ_y; days); and vector density (m), are all climate-dependent variables and calculated using daily-mean temperatures (T; °C) and daily-accumulated rainfall (R; mm day^{-1}) (equations 2-5). b, c, r and τ_x are prescribed climate-independent parameters representing, respectively: the human and mosquito inoculation efficiencies, the human recovery rate (day^{-1}), and the time in humans between infection and infectiousness (the latent period; days). Table S1 shows the prescribed values used for climate-independent model parameters. These prescribed values are taken from [2] and altered for this simplified version of the LMM. The daily vector density, i.e. the number of adult mosquitoes, is proportional to 10-day rainfall accumulations:

$$m_i = \mu_i m_{i-1} + \left(\sum_{d=i-n+1}^{i} R_i \right) \tag{2}$$

where subscript i represents the daily value and n determines the number of days which precipitation is accumulated over (10). The probability of mosquito survival (μ) also impacts the number of adult mosquitoes and is based on a quadratic relationship given with temperature [3]:

$$\mu_i = \begin{cases} -0.0016\,T_i^2 + 0.054\,T_i + 0.45 & \text{if } 0.0 < T_i < 45.0 \\ 0, & \text{otherwise} \end{cases} \tag{3}$$

a and τ_y depend on temperature and are formulated using:

$$a_i = \frac{HBI}{\left(1.0 + \frac{D_g}{(T_i - T_g)}\right)} \tag{4}$$

$$\tau_{y,i} = \frac{D_s}{(T_i - T_s)} \tag{5}$$

where HBI denotes the human blood index (dimensionless); T_g denotes the gonotropic temperature threshold (°C); D_g represents the gonotropic cycle length (° days); T_s denotes the sporogonic temperature threshold (°C); and D_s represents the sporogonic cycle length (° days). This version of the LMM is implemented in *Python* and is freely available [4].

Table S1. Parameter values used for LMM simulations

Parameter	Variable	Value
Human inoculation efficiencies	b	0.5
Mosquito inoculation efficiencies	c	1.0
Human latent period	τ_x	15 days
Human recovery rate	r	0.0284 day^{-1}
Human blood index	HBI	0.5
Gonotrophic threshold temperature	T_g	9.0 °C
Gonotrophic cycle length	D_g	37.0 ° days
Sporogonic threshold temperature	T_s	18 °C
Sporogonic cycle length	D_s	111.0 ° days

S1.2 VECTRI

Within the VECTRI biological model, temperature affects aquatic growth stages of mosquitoes as well as sporogonic and gonotrophic cycles. The effect of precipitation on transmission is represented by a simple, physically-based model of surface pool hydrology whereby the number of available breeding sites depends on rainfall accumulations. Areas of stagnant water decay through evaporation and infiltration, whilst intense rainfall decreases larvae populations through washing away breeding sites. VECTRI also takes into account population density when calculating transmission probabilities, which enables a comparison between urban, peri-urban and rural areas. Further details about the model parameterisation can be found in [5]. The VECTRI source code and documentation can be found at http://users.ictp.it/∼tompkins/vectri/.

S2 Determining the best set of observational climate products

Here we investigate the best combination of precipitation and temperature observations to drive historical malaria simulations. To do this we compare LMM output from nine experiments driven with different precipitation and temperature datasets (Table S2) with estimates of malaria endemicity from MAP (section 2.2). Due to both the LMM and VECTRI requiring precipitation and temperature at a daily temporal resolution, we were limited in our choice of pan-African observational datasets. For our nine LMM experiments we vary between three temperature products: the European Centre for Medium-Range Weather Forecasts (ECWMF) Reanalysis version 5 (ERA5) [6; 7]; Berkeley Earth Surface Temperatures (BEST) [8]; and temperatures from phase 2b of the Inter-Sectoral Impact Model Intercomparison Project (ISIMIP2b) [9], and three precipitation products: ERA5; the Climate Hazards group InfraRed Precipitation with Stations (CHIRPS) [10]; and ISIMIP2b. A description of ERA5 and CHIRPS is provided in the methodology section (section 2.3.1). BEST data uses long-term in situ station data records to output daily temperatures on a 1° latitude/longitude grid. Temperature observations are interpolated onto the horizontal grid using Kriging [11], also known as Gaussian Process Regression, which is the best linear unbiased predictor of the underlying field [8]. Meanwhile, data sources for ISIMIP2b include ERA-Interim reanalysis (ERAI) [12], a WATCH (Water and Global Change) forcing data methodology applied to ERAI data (WFDEI) [13], eartH2Observe forcing data (E2OBS) [14], and NASA/GEWEX (National Aeronautics and Space Administration/Global Energy and Water Exchanges) Surface Radiation Budget data (SRB) [15]. Temperature and precipitation data for ISIMIP2b is outputted on a 0.5° latitude/longitude grid. Data generated for ISIMIP2b aims to support climate-driven

impact-based modelling across a variety of sectors including agriculture, water quality and health. In particular for phase 2b, the project aimed to assess the impacts of an anthropogenic global-mean temperature rise of 1.5°C [9]. All datasets have been remapped onto the same 0.25° latitude/longitude grid as R25 simulations (section 2.3.2) using a first-order conservative interpolation scheme [16].

Table S2 shows the simulation name given to each LMM experiment driven with different climate observational data. The labelling of all experiments follows the same structure with the source of precipitation (P) data followed by the source of temperature (T) observations. We use "E5", "B", "IS" and "Ch" as shorthand to denote ERA5, BEST, ISIMIP and CHIRPS datasets respectively. Figure S1 shows the annual-mean number of days when R_0 is greater than 1.0 in all LMM experiments driven with different observational datasets. In general, all LMM experiments simulate high malaria risk across the Guinea coast, central Africa and coastal regions of south-east Africa. There are substantial differences in predicted malaria risk across parts of equatorial Africa when changing the driving temperature dataset. For example, using temperatures from BEST predicts a much smaller malaria risk across central Africa compared to when using ERA5 or ISIMIP regardless of the chosen precipitation product. Whilst varying the driving temperature dataset leads to substantial differences in LMM-predicted malaria risk, we also conclude that varying the precipitation product can impact simulated malaria incidence. For example, when using precipitation from ISIMIP, simulated malaria risk across coastal regions of western central Africa are much larger compared to when using CHIRPS or ERA5.

To conclude which combination of precipitation and temperature data is best to drive our "observed" malaria simulation experiments, we compute the spatial correlation between the simulated annual-mean number of days when R_0 is greater 1.0 and the estimated Pf incidence rate from MAP. Spatial correlation coefficients between LMM experiments vary between 0.19 to 0.45 (Fig S1). Unsurprisingly, using precipitation from ERA5 has the lowest agreement with MAP-derived incidence rate as ERA5 precipitation relies on parameterisations of deep convection. Spatial correlations improve when using precipitation from EWEMBI due to bias-correcting reanalysis data and the merging of several hydrological products [17]. Using precipitation data from CHIRPS simulates the largest spatial correlations with MAP data regardless of the chosen temperature product. Given that MAP data is partly derived using in situ station and satellite-derived environmental data (section 2.2), it is unsurprising that CHIRPS is the best precipitation product to use. Previous studies have also shown that CHIRPS is one of the most reliable pan-African precipitation products available [10; 18; 19]. However, when considering the best temperature product, temperatures from ERA5 give the largest correlation coefficients regardless of the chosen precipitation dataset. Using temperatures from BEST gives the lowest agreement with MAP estimates which we hypothesise is due to the low number of temperature observations across equatorial Africa [20]. Using temperatures from ISIMIP instead of BEST increases spatial correlations, whilst temperatures from ERA5 increases correlations even further. Higher spatial correlations when using temperatures from ERA5 is unsurprising given it is the only temperature dataset originally produced at a 0.25° horizontal resolution. Given that the combination of CHIRPS precipitation and ERA5 temperatures produces the largest spatial correlation coefficient, for the rest of the study we treat malaria experiments driven with these two datasets as our "observational" malaria experiment.

S3 Simulated temperature and precipitation biases

In this supplementary section we assess the ability of $CP4_h$ and $R25_h$ at simulating historical precipitation and temperature. Given that we conclude that temperatures and

Table S2. LMM malaria transmission experiments using different observational products.

Purpose	Simulation name	Precipitation input data	Temperature input data
Determining the best combination of observational products (LMM only)	E5P_E5T	ERA5	ERA5
	ChP_E5T	CHIRPS	ERA5
	ISP_E5T	ISIMIP	ERA5
	E5P_BT	ERA5	BEST
	ChP_BT	CHIRPS	BEST
	ISP_BT	ISIMIP	BEST
	E5P_IST	ERA5	ISIMIP
	ChP_IST	CHIRPS	ISIMIP
	ISP_IST	ISIMIP	ISIMIP

precipitation from ERA5 and CHIRPS are the best sources of driving climate data for historical LMM experiments (section S2), we evaluate climate simulations using these two datasets. Similar conclusions are reached when using other temperature or precipitation datasets. Consistent with previous studies [21; 22], parameterised convection favours the occurrence of light rainfall (Fig S2a-b, S2d-e, and S3a). However, even with more frequent light rainfall days in $R25_h$ compared to $CP4_h$, errors in 10-day precipitation accumulations are greater in $CP4_h$ particularly over high-altitude regions such as the East African highlands and Mount Cameroon (Fig S2g,h). For example, the RMSD in 10-day precipitation accumulations is 1.64 mm greater in $CP4_h$ than $R25_h$. Figure S3a shows the fractional contribution of all daily precipitation rates across land points south of 20°N. We find a larger fraction of days with light precipitation ($\leq$ 10 mm day^{-1}) in $R25_h$ compared to $CP4_h$. However, $CP4_h$ overestimates the fraction of days when precipitation is greater than approximately 20 mm day^{-1} to a greater degree than $R25_h$. Therefore, we conclude that larger biases in 10-day precipitation accumulations in $CP4_h$ compared to $R25_h$ (Fig S2i) are associated with too many heavy rainfall days.

$CP4_h$ has a strong negative temperature bias across the Sahel (Fig S2j). Decomposing annual-mean errors into seasonal contributions highlights that similar errors are found across the Sahel during dry seasons. From July to September, the wet season associated with the West African monsoon, near-surface temperature biases are minimum (not shown). Given that temperature errors across the Sahel are largest during dry seasons, and that malaria transmission is favoured during wet conditions (section 2.1), we also investigate wet-day temperature biases (Fig S2m-o). In general, $CP4_h$ has larger wet-day temperature errors with the RMSD being 0.18°C greater in comparison with $R25_h$. The difference in wet-day temperatures between $CP4_h$ and $R25_h$ highlights that temperatures are consistently cooler in $CP4_h$ when it is raining (Fig S2o). Cooler wet-day temperatures in $CP4_h$ compared to $R25_h$ is seen across all seasons (not shown). This is consistent with findings by [22] who conclude that higher cloud tops in $CP4_h$ leads to a greater reflection of incoming shortwave radiation and reduced near-surface heating. Figure S3b shows the fractional contribution of temperatures across all land grid points. Consistent with aforementioned results (Fig S2j-o), $CP4_h$ favours cooler near-surface temperatures than R25. Outside of approximately 21 to 29°C, $R25_h$ is more consistent with observations compared to $CP4_h$. This indicates that R25 better resolves the frequency of high near-surface temperatures ($\geq$ 29°C). To summarise, whilst the rainfall frequency and daily-mean precipitation rate is better resolved in $CP4_h$ compared to $R25_h$, larger errors in 10-day precipitation accumulations and near-surface temperatures are found in $CP4_h$. In this study, we investigate the impact of cooler wet-day temperatures and higher 10-day

rainfall accumulations in $CP4_h$ compared to $R25_h$ on simulated malaria transmission.

S4 Supplementary figures

Fig S1. Annual-mean number of days when R_0 is greater 1.0 from LMM experiments driven with different observational products. First, second and third rows are driven with ERA5, CHIRPS and ISIMIP precipitation respectively. Whilst first, second and third columns are driven with ERA5, BEST and ISIMIP temperature. In all panels boxed values note the spatial correlation coefficient between the annual-mean number of days when R_0 is greater 1.0 and MAP data (Fig 1a). To ensure that the spatial correlation is not biased towards regions of low malaria incidence, we remove all grid points where the MAP-derived Pf incidence rate is smaller than 0.1. We also removed grid points where the simulated annual-mean number of days when R_0 is greater than 1.0 is outside the range of 15.0 and 140.0. To be consistent with the time span of available MAP data [23], we only compare malaria model output which is driven with climate model data from years 2000 to 2007. All correlations are statistically significant at a 99% confidence interval. Land and country boundaries were added using Natural Earth; free vector and raster map data available at naturalearthdata.com.

Fig S2. Annual-mean differences in (a-c) 10-day precipitation accumulations (mm), (d-f) the number of wet days ($\geq$ 1 mm), (g-i) mean wet-day precipitation rate (mm), (j-l) daily-mean near-surface air temperature (°C), and (m-o) daily-mean wet-day near-surface air temperature (°C). Differences are shown between (first column) $CP4_h$ and observations, (second column) $R25_h$ and observations, and (third column) $CP4_h$ and $R25_h$. Values above each panel label, document the root mean squared difference (RMSD) across land points south of 20°N in each panel. Land and country boundaries were added using Natural Earth; free vector and raster map data available at naturalearthdata.com.

Fig S3. Fractional contributions of (a) daily-accumulated precipitation rates (mm day^{-1}) and (b) daily-mean near-surface air temperatures (°C) across all land points south of 20°N in bins of 2 mm day^{-1} and 1°C for (orange) $CP4_h$, (blue) $R25_h$, and (grey) observations. In (a) a subset panel zooms into the fractional contributions of daily-accumulated precipitation rates up to 10 mm day^{-1}. A light grey rectangle in panel (a) denotes the area of focus.

References

1. Jones AE, Wort UU, Morse AP, Hastings IM, Gagnon AS. Climate prediction of El Niño malaria epidemics in north-west Tanzania. Malaria journal. 2007; 6(1): 1–15.

2. Ermert V, Fink AH, Jones AE, Morse AP. Development of a new version of the Liverpool Malaria Model. I. Refining the parameter settings and mathematical formulation of basic processes based on a literature review. Malaria journal. 2011; 10(1): 1–17.

3. Martens W, Jetten T, Rotmans J, Niessen L. Climate change and vector-borne diseases: a global modelling perspective. Global environmental change. 1995; 5(3): 195–209.

4. Jones A, J AZ, S K. C2MA-workshop/c2ma-tutorials: c2ma post workshop; 2021. Available from: https://doi.org/10.5281/zenodo.5494445.

5. Tompkins AM, Ermert V. A regional-scale, high resolution dynamical malaria model that accounts for population density, climate and surface hydrology. Malaria journal. 2013; 12(1): 1–24.

6. Copernicus Climate Change Service (C3S). ERA5: Fifth generation of ECMWF atmospheric reanalyses of the global climate. Copernicus Climate Change Service Climate Data Store (CDS). 2017.

7. Hersbach H, Bell B, Berrisford P, Hirahara S, Horányi A, Muñoz-Sabater J, et al. The ERA5 global reanalysis. Quart J Roy Meteor Soc. 2020; 146(730): 1999–2049.

8. Rohde R, Muller R, Jacobsen R, Muller E, Perlmutter S, Rosenfeld A, et al. A new estimate of the average Earth surface land temperature spanning 1753 to 2011. Geoinfor Geostat: An Overview 1: 1. 2013.

9. Frieler K, Lange S, Piontek F, Reyer CP, Schewe J, Warszawski L, et al. Assessing the impacts of 1.5 C global warming–simulation protocol of the Inter-Sectoral Impact Model Intercomparison Project (ISIMIP2b). Geoscientific Model Development. 2017; 10(12): 4321–4345.

10. Funk C, Peterson P, Landsfeld M, Pedreros D, Verdin J, Shukla S, et al. The climate hazards infrared precipitation with stations—a new environmental record for monitoring extremes. Sci Data. 2015; 2(1): 1–21.

11. Cressie N. The origins of kriging. Mathematical geology. 1990; 22: 239–252.

12. Dee DP, Uppala SM, Simmons AJ, Berrisford P, Poli P, Kobayashi S, et al. The ERA-Interim reanalysis: Configuration and performance of the data assimilation system. Quarterly Journal of the royal meteorological society. 2011; 137(656): 553–597.

13. Weedon GP, Balsamo G, Bellouin N, Gomes S, Best MJ, Viterbo P. The WFDEI meteorological forcing data set: WATCH Forcing Data methodology applied to ERA-Interim reanalysis data. Water Resources Research. 2014; 50(9): 7505–7514.

14. Dutra E. Report on the current state-of-the-art Water Resources Reanalysis, Earth2observe deliverable no. D. 5.1; 2015.

15. Stackhouse Jr PW, Gupta SK, Cox SJ, Zhang T, Mikovitz JC, Hinkelman LM. The NASA/GEWEX surface radiation budget release 3.0: 24.5-year dataset. Gewex news. 2011; 21(1): 10–12.

16. Jones PW. First-and second-order conservative remapping schemes for grids in spherical coordinates. Monthly Weather Review. 1999; 127(9): 2204–2210.

17. Lange S. EartH2Observe, WFDEI and ERA-Interim data Merged and Bias-corrected for ISIMIP (EWEMBI). V. 1.1. 2019.

18. Beck HE, Vergopolan N, Pan M, Levizzani V, Van Dijk AI, Weedon GP, et al. Global-scale evaluation of 22 precipitation datasets using gauge observations and hydrological modeling. Hydrology and Earth System Sciences. 2017; 21(12): 6201–6217.

19. Muthoni FK, Odongo VO, Ochieng J, Mugalavai EM, Mourice SK, Hoesche-Zeledon I, et al. Long-term spatial-temporal trends and variability of rainfall over Eastern and Southern Africa. Theoretical and Applied Climatology. 2019; 137(3): 1869–1882.

20. Rohde R, Muller R, Jacobsen R, Perlmutter S, Rosenfeld A, Wurtele J, et al. Berkeley earth temperature averaging process, geoinfor. geostat.-an overview, 1, 2. Geoinformatics Geostatistics An Overview. 2013; 1(2): 20–100.

21. Marsham JH, Dixon NS, Garcia-Carreras L, Lister GM, Parker DJ, Knippertz P, et al. The role of moist convection in the West African monsoon system: Insights from continental-scale convection-permitting simulations. Geophysical Research Letters. 2013; 40(9): 1843–1849.

22. Stratton RA, Senior CA, Vosper SB, Folwell SS, Boutle IA, Earnshaw PD, et al. A Pan-African convection-permitting regional climate simulation with the met office unified model: CP4-Africa. Journal of Climate. 2018; 31(9): 3485–3508.

23. Hay SI, Snow RW. The Malaria Atlas Project: developing global maps of malaria risk. PLoS medicine. 2006; 3(12): e473.
