# Supplementary figures and images for: The effect of explicit convection on simulated malaria transmission across Africa
Source: PLoS One. 2024 Apr 16;19(4):e0297744. doi: 10.1371/journal.pone.0297744 (PMC11020401; doi:10.1371/journal.pone.0297744)

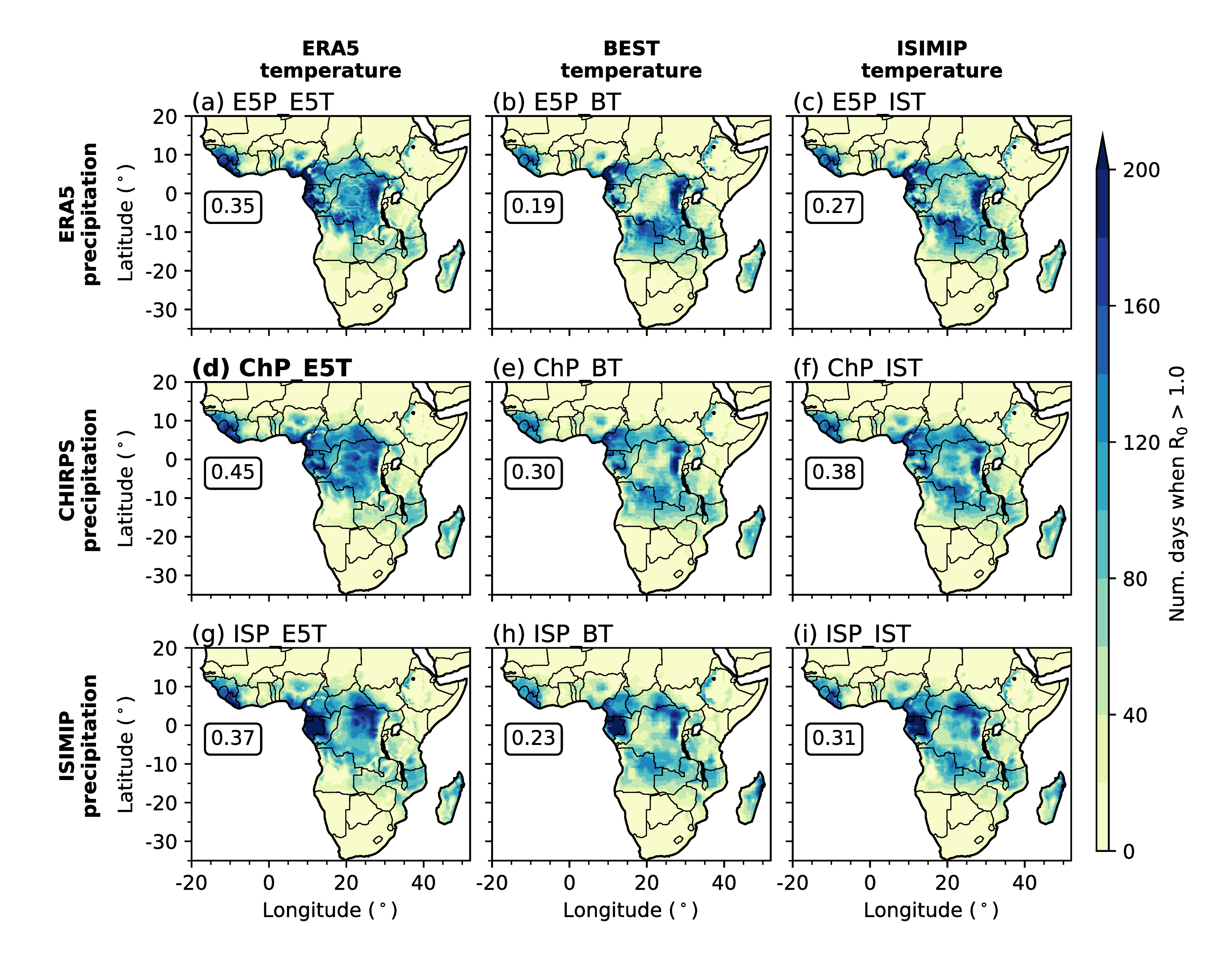

Supplement: S1 Fig — First, second and third rows are driven with ERA5, CHIRPS and ISIMIP precipitation respectively. Whilst first, second and third columns are driven with ERA5, BEST and ISIMIP temperature. In all panels boxed values note the spatial correlation coefficient between the annual-mean number of days when R0 is greater 1.0 and MAP data (Fig 1a). To ensure that the spatial correlation is not biased towards regions of low malaria incidence, we remove all grid points where the MAP-derived Pf incidence rate is smaller than 0.1. We also removed grid points where the simulated annual-mean number of days when R0 is greater than 1.0 is outside the range of 15.0 and 140.0. To be consistent with the time span of available MAP data [62], we only compare malaria model output which is driven with climate model data from years 2000 to 2007. All correlations are statistically significant at a 99% confidence interval. Land and country boundaries were added using Natural Earth; free vector and raster map data available at naturalearthdata.com. (TIF) [file pone.0297744.s002.tif]

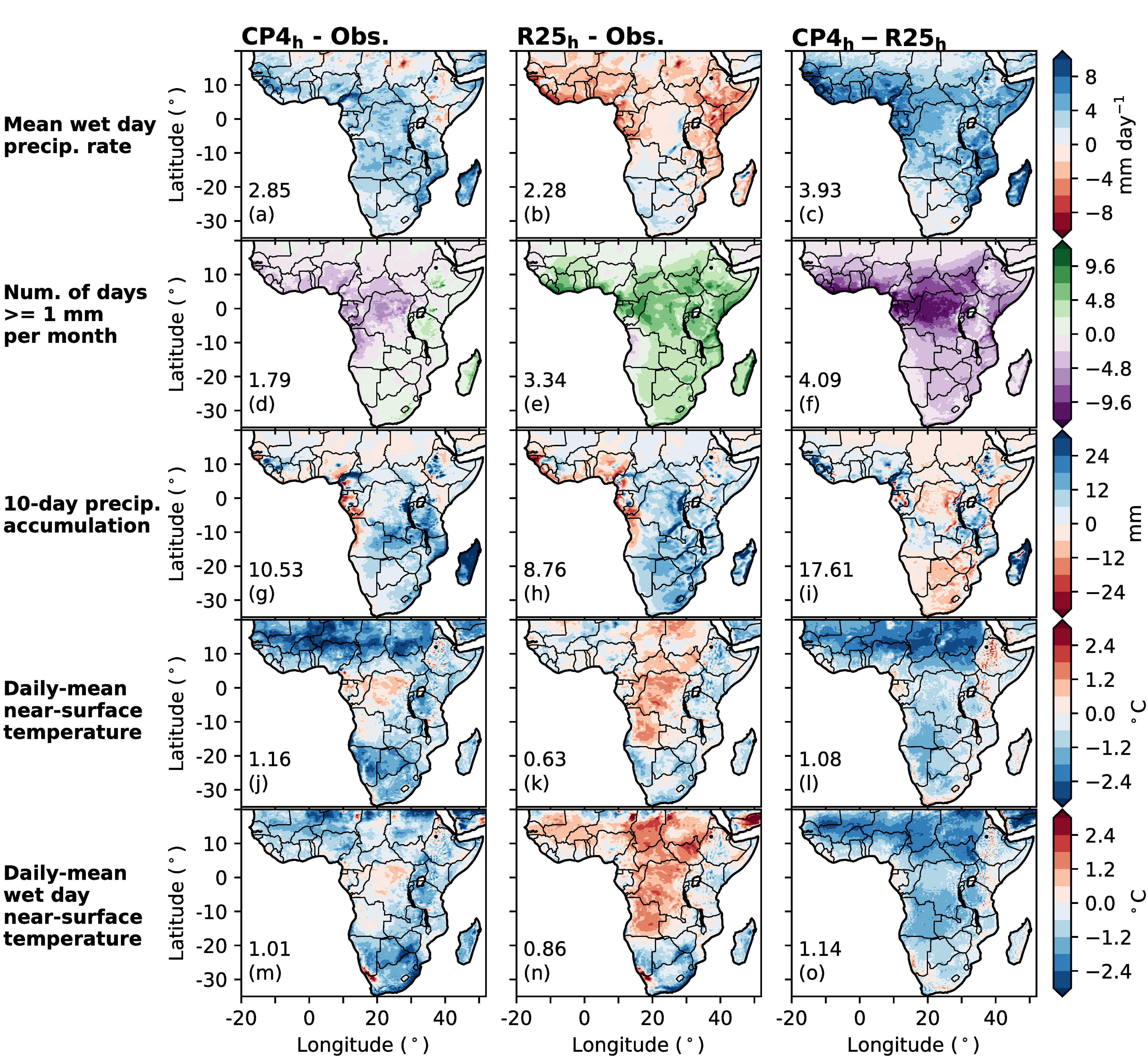

Supplement: S2 Fig — Annual-mean differences in (a-c) 10-day precipitation accumulations (mm), (d-f) the number of wet days (≥ 1 mm), (g-i) mean wet-day precipitation rate (mm), (j-l) daily-mean near-surface air temperature (°C), and (m-o) daily-mean wet-day near-surface air temperature (°C). Differences are shown between (first column) CP4h and observations, (second column) R25h and observations, and (third column) CP4h and R25h. Values above each panel label, document the root mean squared difference (RMSD) across land points south of 20°N in each panel. Land and country boundaries were added using Natural Earth; free vector and raster map data available at naturalearthdata.com. (TIF) [file pone.0297744.s003.tif]

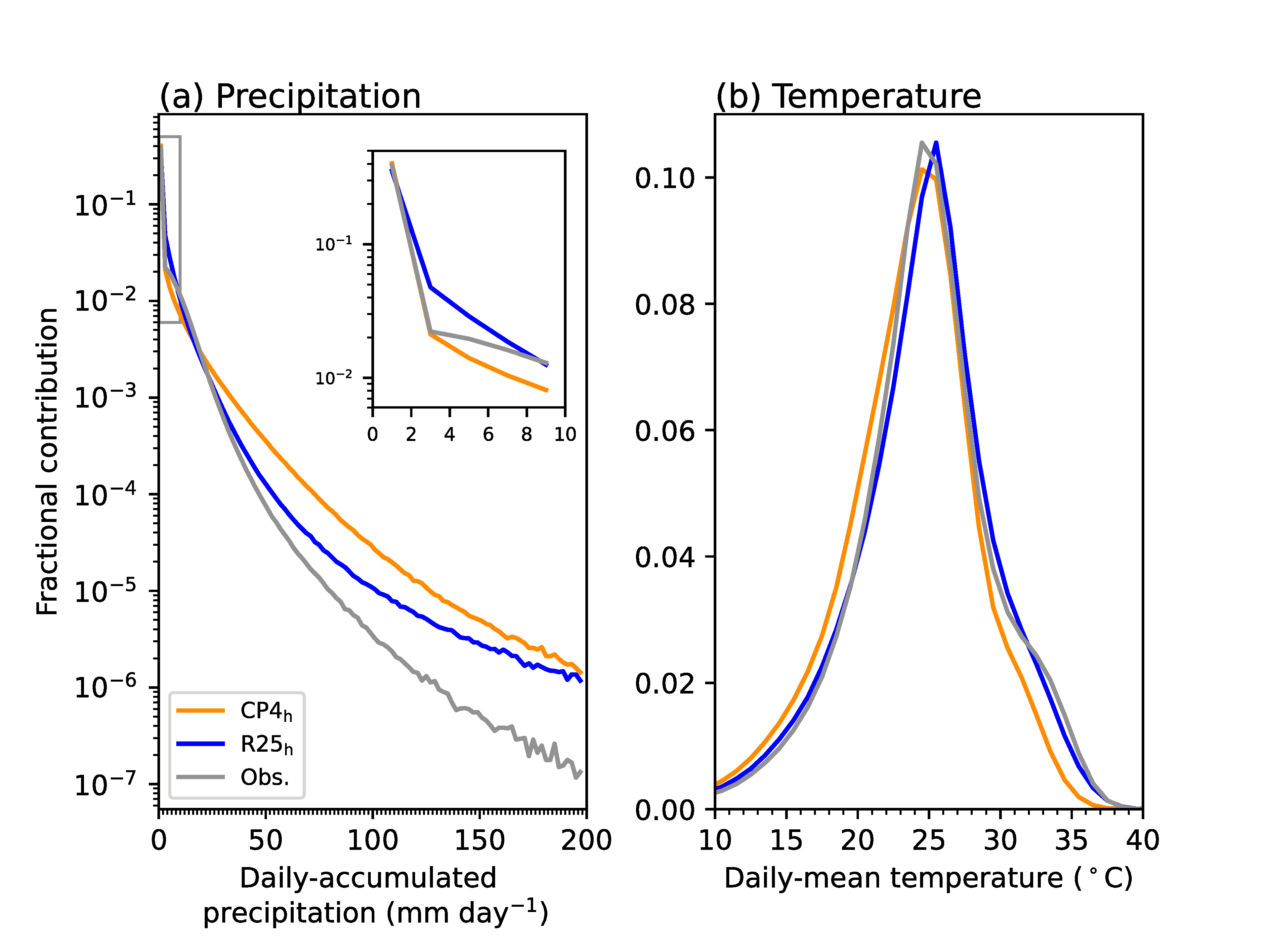

Supplement: S3 Fig — Fractional contributions of (a) daily-accumulated precipitation rates (mm day−1) and (b) daily-mean near-surface air temperatures (°C) across all land points south of 20°N in bins of 2 mm day−1 and 1°C for (orange) CP4h, (blue) R25h, and (grey) observations. In (a) a subset panel zooms into the fractional contributions of daily-accumulated precipitation rates up to 10 mm day−1. A light grey rectangle in panel (a) denotes the area of focus. (TIF) [file pone.0297744.s004.tif]
