# Supplementary material for: The effect of explicit convection on simulated malaria transmission across Africa
Source: PLoS One. 2024 Apr 16;19(4):e0297744. doi: 10.1371/journal.pone.0297744 (PMC11020401; doi:10.1371/journal.pone.0297744)
Supplement: Attachment — Submitted filename: response_to_reviewers_NOV23.docx [file pone.0297744.s005.docx]

*The effect of explicit convection on simulated malaria transmission across Africa*

*Submitted*

*We would like to thank both reviewers for their time critiquing our manuscript. The comments provided highlighted a need to refine conclusions and clarify performed malaria experiments. Our response to comments are shown in italics, with* ***bold italics*** *indicating updated/edited manuscript text. We have also provided a manuscript copy with tracked changes. We look forward to receiving your comments on our responses and we hope we have clarified all issues.*

*Talib et al.*

Reviewers' comments:

**Comments to the Author**

Reviewer #1: SUMMARY: The authors compare the output of malaria model simulations for sub-saharan Africa when convection is or is not explicitly modeled in a climate dataset. They compare these outputs to those of the MAP malaria data to estimate their accuracy. They then use the two climate datasets for future predictions of malaria to compare how predictions change as a function of accounting for convection. They find that the influence of convection on predictions depends on the model (VECTRI is driven more by changes in extreme precipitation, while LMM is more sensitivity to changes in temperature) and the region of interest. They also find that future malaria predictions are most sensitive to changes in temperature due to convection.

Overall, I think it is interesting study with a well-written manuscript. The majority of my suggestions are minor and focus on improving the structure of how the methods and results are presented. The authors are free to take them into account or not, but I do think clarifying how the different combinations of models and data sources are referred to will increase the manuscript's usability.

STRENGTHS:

The question of which climate data we are using, and how it interacts with the choice of model, is an important question and will be of interest to disease modelers.

The analyses conducted are very comprehensive, containing multiple levels of sensitivity analyses.

The manuscript is well written, and the discussion is very thorough.

*We would like to thank reviewer 1 for their time and constructive comments towards our manuscript. We have taken our time to provide an extensive response to all comments which we hope meet the reviewer’s expectations.*

MAJOR SUGGESTIONS:

I found the presentation of the methods and results a bit difficult to follow due to the many sensitivity analyses conducted. I make some small suggestions below that could help with this. I also suggest the authors use some form of sub-titles as signposts to help guide the reader to more easily orient themselves to which sensitivity analysis or comparison is currently being addressed. It may also help to divide the figures into ones corresponding to each sensitivity analysis (although I recognize there may be a limit on figures).

*As explained below, we have made changes to the manuscript to improve readability of the malaria experiments performed. These changes include paragraph headings in section 2.4 and a new formatted table 2. We also ensured that paragraphs are partitioned by the set of malaria experiments being discussed. We decided against partitioning figures into specific sensitivity experiments. Doing so would have generated several more figures. It is also helpful for the reader to easily compare between sensitivity experiments.*

line 338: The conclusion that future changes in malaria transmission are primarily due to temperature seems to me that it could also be an artefact of the model used given that LMM is sensitive to changes in temperature input. Some discussion on this should be added and it should be mentioned that this finding is dependent on the model used, and not a generalization that climate change driven changes in rainfall will not impact malaria transmission.

*Whilst we do not discuss the sensitivity of predicted malaria changes to climate-dependent malaria model parameterisations when introducing results from LMM sensitivity experiments (lines 352 - 354), we do discuss that predicted future changes in malaria transmission are sensitive to malaria model parameterisations in other parts of the paper. We discuss this important conclusion in: the end of section 3.3; the fourth discussion paragraph; and in the second conclusion paragraph. In light of this comment we have added the phrase “LMM-simulated” to line 338 so it reads,* ***From these sensitivity experiments we conclude that changes in LMM-simulated malaria transmission are mainly associated with temperature changes for both CP4 and R25 configurations****.*

Some further discussion and qualification is needed about using the MAP data as "observed" data, given it is itself the result of a geo-statistical model based on climate variables. Specifically, it may be helpful to mention the variables used in that model (Worldclim for precipitation and MODIS for temperature), how those relate to the two climate models used here, and whether we may a priori expect one to perform better than the other because of this.

*In section 2.2 we now provide further details regarding the environmental variables used to derive the MAP statistical model:* ***Focusing on environmental predictors, MAP utilises precipitation from WorldClim, which interpolates thousands of station observations (Fick et al., 2017), as well as surface temperature, wetness and land cover from Terra and Aqua Moderate Resolution Imaging Spectroradiometer (MODIS) satellite products (Wan et al., 2002).***

*Further discussion of utilised environmental variables for generating MAP data allows us to conclude that no single climate model configuration will be more favourable. We have also added the following sentence to section 2.2:* ***Given that MAP output is only derived using observations of environmental conditions rather than climate model data, we cannot postulate that malaria model output driven by a certain climate model configuration will have a stronger agreement with MAP data.***

MINOR SUGGESTIONS:

Given the wide readership of PLoS One and the audience of this paper, it would be helpful to add a sentence or two further defining convection and the expected impact of it on the study area (like you do in line 420).

*Good point. We now provide a brief explanation of convection in the abstract (4^th^ line) and introduction (line 53-54).*

line 151: It may be helpful to state explicitly here that CP4 represents a model with convection explicitly modeled and R25 represents the "baseline" model not considering convection (or parameterizing it).

*Added the following sentence to line 162:* ***In this study R25 is treated as the ``baseline" climate model, whilst CP4 is used to investigate the impact of explicitly representing convection on malaria transmission.***

line 192: It may help to make this section clearer by splitting each sensitivity analysis into its own paragraph and adding a topic sentence specifically stating what question that sensitivity analysis is meant to address. I found myself getting a bit lost between the different analyses.
line 196: I found the labeling system a bit hard to follow. One suggestion could be to split Table 2 up by sensitivity analysis or experiment, to make it clearer what combinations of data sources are addressing what scientific questions. It just becomes hard to quickly see what the difference is between two combinations of variables elsewhere in the paper, especially in Figure 2 and 4.

*Thank you for these two comments regarding readability of malaria model experiments performed. Given the number of malaria transmission integrations, understanding the purpose of different sensitivity experiments may be challenging to understand after an initial read. In light of these comments, we have restructured table 2 and added paragraph headers to section 2.4. The updated table partitions sensitivity experiments by their main objective, whilst paragraph headers support the reader’s understanding of the purpose of sensitivity experiments discussed. Given this comment, we have also changed the format of table S2, which summarises LMM experiments performed to determine the best combination of observational products.*

Fig 2: It is a bit confusing having the color bar positive values correspond to blue and negative to red, as the norm is the opposite. Also please state in the figure caption what the numbers in boxes correspond to.

*We have changed the colour bar in figures 2 and 4 so positive and negative values correspond to red and blue respectively. We had already stated what the boxed values corresponded to, however, we have now changed the figure caption to improve clarity. The figure caption now includes:*

***In each panel, boxed values document the root mean squared difference across land points south of 20°N.***

*In light of this comment, we have also clarified what boxed values denote in figures 4 and S1.*

line 201: To reduce confusion and the number of different sensitivity analyses and comparisons, I would move this experiment to the supplement, or make it the main default (See below).
line 284: If the bias-corrected data is a better fit, it seems like that should be used in the primary analysis and the findings comparing non-corrected and corrected in a supplement. Or is the standard in climatology to present the non-bias corrected data? If you choose to keep the non bias-corrected data as the main analysis, I would move the section on bias-corrected to the supplement (see above).

*In this study we wanted to underline the added value of using convection-permitting climate model data. Therefore, we have decided to leave the manuscript as it stands and include experiments driven by bias-corrected and non-bias corrected climate model data. Calibrating both explicit and parameterised convection simulations will bring both climate models closer to observations, thereby minimizing differences between different climate simulations. Solely relying on bias-corrected simulations also introduces a requirement to consider the best bias-correcting technique, which is out of scope of this study. Including malaria simulations driven with bias-corrected data allows us to be transparent regarding the sources of simulation differences.*

Figure 4: Is there another way you could describe or present panels c,f,i,l? The current labeling system takes some time to interpret and is not easy to understand. Or maybe add a title to this column as you did for the rows?

*We have added labels to each column to improve clarity regarding comparisons between changes when using explicit and parameterised experiments (c,f,i and l).*

line 373: These first two sentences could be split up to help with readability. Also the sentence beginning on line 397.

*We have split the first two sentences of the first discussion paragraph to improve readability. For line 397, we have shortened the sentence.*

line 535: This link seems to be broken. I think it is missing the tilde (~)

*We have added a tilde to the manuscript. An error occurred due compiling of manuscript.*

*End of review 1*

Reviewer #2: The authors examine the difference in the representation of annual mean malaria in Africa in two malaria models; LMM and VECTRI. The models include the influence of meteorology on disease transmission, and the main goal of the study is to assess sensitivity to the meteorological data used to force the model. The data sets tested are output from two regional configurations of the UK Met Office Unified Model (MetUM); one with a convection-permitting 4.5km grid that explicitly represents convection, and the other with a more standard convective parameterization scheme at a horizontal grid spacing of 25km. The main conclusion presented is that the 4.5km model data provides better agreement with the observations and that "future work should utilize high-resolution explicit-convection climate simulations" as a result.

Malaria is one of the most pressing health issues in the world, and understanding how transmission will potentially evolve under climate change is critical. Unfortunately, I have serious reservations about the methodology of the study and its main conclusion. I recommend that the manuscript be returned to the authors for Major and mandatory revisions prior to publication

*We would like to thank reviewer 2 for their time and constructive comments towards our manuscript. We have taken our time to provide an extensive response to both major concerns. Through doing so, the clarity of the manuscript has improved and provides a more accurate discussion of simulated outputs. We were wrong to conclude that only convection-permitting simulations should be used in the foreseeable future. We have changed the majority of our conclusive statements.*

Major Concerns

As noted above, the manuscript presents as its major conclusion that using a climate model with explicit convection to drive the malaria model provides superior results to a conventional physics parameterization, and as such "future work should utilize high-resolution explicit-convection climate simulations" (Abstract, last line). However, the evidence presented falls well short of proving or even fully supporting this hypothesis. In Figure 1, the authors do show that there is an improvement in the pattern correlation of annual mean malaria Ro for the LMM for the period 2000-2007 when the convective-permitting configuration (CP4) is used instead of the conventional model (R 25). However, this result a relatively small difference in pattern correlation (0.44 vs 0.37) for a relatively short period, for a single ensemble member of a single climate model for a single malaria model. For the VECTRI model, also presented in Figure 1, the skill is literally identical for CP4 and R25. Given these caveats I do not believe that these results can be generalized to the conclusions the manuscript presents.

*There are two key results which led us to conclude that using data from convection-permitting simulations is more reliable to predict malaria transmission than using parameterised simulations: (1) As mentioned by the reviewer, LMM spatial correlations with MAP data is greater when using CP4 data compared to R25 (figure 1); (2) Root-mean squared differences (RMSDs) between malaria models driven with observations and climate model data are lower when using data from CP4 compared to R25 for both LMM and VECTRI (figure 2b, c, j and k). Regarding (1), spatial correlations were calculated using approximately 24,000 grid points. Hence, even a small change in correlation is meaningful given the large sample size. With regards to (2), we were transparent regarding the magnitude of relative changes in RMSD. For the LMM, a RMSD reduction of 23% when using the LMM is still a significant improvement. However, the reviewer is correct to raise concerns about this conclusion given than VECTRI spatial correlations with MAP data is similar when using both CP4 and R25 data (figure 1), and, bias-correcting CP4 and R25 data substantially reduces differences in RMSDs between LMM experiments driven with observations and model data. In light of this, we have changed several conclusion statements in the manuscript and ensured we are more explicit regarding the uncertainties in concluding that explicitly representing convection improves simulated malaria. For example in the abstract we now include:*

***Even though explicitly representing convection improves rainfall characteristics, concluding that explicit convection improves simulated malaria transmission depends on the chosen metric and malaria model.***

*We also acknowledge that the time integration and the use of a single climate model is a limitation. This point was honestly discussed in the last discussion paragraph. Our* *results fit to our numerical set up and cannot be generalized to all convective permitting and regional climate model simulations.*

The results presented in Figure 2 also call this main conclusion into question. Here the manuscript assesses the impact of temperature and precipitation independently, as well as the effect of climatological biases. Rather than confirming the inherent advantages of the CP4 integrations, Figs. 2g and 2h show that when the climatological biases are removed from each model's output that the R25 actual provides a better comparison to observations. The manuscript notes this result on page 12, stating that "disparities between simulated malaria transmission are predominantly associated with mean climatic conditions". This is a significantly different and less novel conclusion than the claim that explicitly resolving convection leads to a better simulation of malaria transmission. While it is certainly possible, and hoped for, that a more physically accurate representation of convection will lead to a smaller mean bias it is by no means assured.

*Bias-correcting climate model data pushes both malaria simulations closer to the reference control run which is preformed using observations. Solely relying on bias-corrected simulations misrepresents the full improvement when using convection-permitting simulations. This is why we present non-calibrated results as they truly represent the added value of using high resolution convection permitting simulations. We agree that more work is required on bias-correcting, for example comparing different methods such as CDF-t., quantile correction, weather generators, but this is out of scope of the paper and would require a lot of effort and funding on its own. In light of reduced differences in malaria experiments when driving with bias-corrected data, we have changed all conclusive statements within the manuscript. For example, we have included the following line in the abstract*:

***Whilst we conclude improvements of 45% and 23% in root mean squared differences of the annual-mean reproduction number for VECTRI and the LMM respectively, bias-correcting mean climate conditions minimises these improvements.***

6. PLOS authors have the option to publish the peer review history of their article ([what does this mean?](https://journals.plos.org/plosone/s/editorial-and-peer-review-process#loc-peer-review-history)). If published, this will include your full peer review and any attached files.


If you choose “no”, your identity will remain anonymous but your review may still be made public.


**Do you want your identity to be public for this peer review?** For information about this choice, including consent withdrawal, please see our [Privacy Policy](https://www.plos.org/privacy-policy).

Reviewer #1: No

Reviewer #2: No
